# Accumulation of Arachidonic Acid, Precursor of Pro-Inflammatory Eicosanoids, in Adipose Tissue of Obese Women: Association with Breast Cancer Aggressiveness Indicators

**DOI:** 10.3390/biomedicines10050995

**Published:** 2022-04-26

**Authors:** Lobna Ouldamer, Marie-Lise Jourdan, Michelle Pinault, Flavie Arbion, Caroline Goupille

**Affiliations:** 1 Department of Gynecology, Centre Hospitalier Régional Universitaire de Tours, Hôpital Bretonneau, 2 Boulevard Tonnellé, 37044 Tours, France; lobna.ouldamer@univ-tours.fr; 2 Laboratoire N2C, Nutrition, Croissance et Cancer, INSERM, UMR1069, Université de Tours, 10 Boulevard Tonnellé, 37044 Tours, France; ml_jourdan@yahoo.fr (M.-L.J.); michelle.pinault@univ-tours.fr (M.P.); 3 Department of Pathology, Centre Hospitalier Régional Universitaire de Tours, Hôpital Bretonneau, 2 Boulevard Tonnellé, 37044 Tours, France; f.arbion@chu-tours.fr

**Keywords:** obesity, breast cancer, body mass index, long-chain polyunsaturated fatty acid n-6, arachidonic acid, fatty acids, adipose tissue

## Abstract

While obesity is linked to cancer risk, no studies have explored the consequences of body mass index (BMI) on fatty acid profiles in breast adipose tissue and on breast tumor aggressiveness indicators. Because of this, 261 breast adipose tissue samples of women with invasive breast carcinoma were analyzed. Fatty acid profile was established by gas chromatography. For normal-weight women, major changes in fatty acid profile occurs after menopause, with the enrichment of long-chain polyunsaturated fatty acids (LC-PUFAs) of both n-6 and n-3 series enrichment, but a stable LC-PUFAs n-6/n-3 ratio across age. BMI impact was analyzed by age subgroups to overcome the age effect. BMI increase is associated with LC-PUFAs n-6 accumulation, including arachidonic acid. Positive correlations between BMI and several LC-PUFAs n-6 were observed, as well as a strong imbalance in the LC-PUFAs n-6/n-3 ratio. Regarding cancer, axillary lymph nodes (*p* = 0.02) and inflammatory breast cancer (*p* = 0.08) are more frequently involved in obese women. Increased BMI induces an LC-PUFAs n-6 accumulation, including arachidonic acid, in adipose tissue. This may participate in the development of low-grade inflammation in obese women and breast tumor progression. These results suggest the value of lifestyle and LC-PUFAs n-3 potential, in the context of obesity and breast cancer secondary/tertiary prevention.

## 1. Introduction

Accumulating evidence has linked obesity to cancer risk. Thus, obesity is identified as a convincing risk factor or probable factor in postmenopausal breast, endometrial, ovary, colorectal or aggressive prostate cancers [1]. In addition, the association between obesity and cancer recurrence or overall survival of the cancer patient is suspected, particularly in breast, colon, prostate and other cancers [2,3,4]. Interestingly, epidemiological studies, including weight loss by diet control and/or increased physical activity, have suggested that negative factors linked to obesity may be partly reversible, concerning cancer risk or cancer survival [5,6]. Pathologies associated with obesity, such as diabetes, dyslipidaemia and increased blood pressure, or a relative under-dosing of chemotherapy, may directly affect the survival of obese cancer patients. However, the mechanisms that link obesity with cancer incidence or promote its progression remain to be resolved [7]. 

Bioactive mediators can be metabolized from some fatty acids, mainly arachidonic acid [AA, 20:4n-6], eicosapentaenoic acid [EPA, 20:5n-3] and docosahexaenoic acid [DHA, 22:6n-3]. According to preclinical and clinical studies, the polyunsaturated fatty acids (PUFAs) n-6 and n-3 are believed to have opposite effects. PUFAs n-6 are associated with inflammation and cancer development, whereas PUFAs n-3 have anti-inflammatory and anti-cancer properties [8,9,10]. In agreement with this, an increase in the PUFA n-6/n-3 ratio or a deficiency in some PUFA n-3 content is associated with breast cancer risk [11,12] or with aggressiveness/progression indicators, such as tumor multifocality, a tumor feature linked to early relapse [13] or bone metastases associated with breast tumors [14]. In addition, obesity status and low PUFA n-3 content are two factors associated with inflammatory breast tumors, a breast tumor phenotype with a worse prognosis [15,16,17]. 

Adipose tissue has an essential function in the storage of triglycerides and may be considered a long-term biomarker, reconstructing exposure to dietary lipids over the last 12 months [18]. However, Bolton-Smith et al. [19] highlighted an age-dependent remodeling of the fatty acid profile, independent of diet, with an increased content of long-chain PUFAs (LC-PUFAs) n-6 and n-3, which suggests an adipose metabolism changing with aging and menopausal status. In recent years, several studies have reported that adipose tissue structure and endocrine functions are modified by age [20] and also by obesity [21]. Several similar points between obesity and aging have been reported for adipose tissue with hypertrophic adipocytes, excessive deposition of extracellular matrix, increased infiltration of inflammatory macrophages and chronic low-grade inflammation [22]. 

Currently, studies have not examined the association between the fatty acid profile of breast adipose tissue and body mass index (BMI) in the breast cancer context. The hypothesis of this work was that increased BMI might modify the fatty acid profile of adipose tissue and be associated with indicators of breast tumor aggressiveness. This retrospective study was based on 261 breast cancer patients, cared for in Tours University Hospital (central region of France).

## 2. Materials and Methods

### 2.1. Population and Samples

All patients had clinical care at the Tours University Hospital (France). Adipose tissue samples were collected during surgery for the tumor resection. Adipose tissue was collected in a zone considered as healthy, beyond the margin that is defined when the breast tumor mass was excised. A previous study showed that the composition of the adipose tissue in this zone is not altered by the tumor [23]. Samples were stored in liquid nitrogen in our university hospital cryobank. 

The research protocol was approved by the Institutional Review Board (IRB) of the teaching hospital of Tours (France) and the biological collection was declared to the French ministry of Health (reference No. DC-2008-308). As our study was non-interventional and retrospective, formal written consent from patients included was not necessary.

The population included women with invasive breast cancer (*n* = 261) who had breast surgery between January 2009 and June 2012. This retrospective population was previously described [14]. Briefly, we included all adipose tissues associated with less frequent phenotypes (negative hormone-receptor tumors: triple negative and HER2+++ tumors), and patients with positive hormone-receptor tumor phenotypes (luminal A and B) were selected during the same period and chosen to match the age distribution of the tumor-carrying patient groups. The breast adipose tissue collection cryobank was authorized and declared to the French Ministry of higher Education and Research (reference No. DC-2008-308), and the study was approved by the Tours University Teaching Hospital Review Board. 

Clinical information was collected from medical records and included demographic characteristics (age, BMI), presence or not of pathologies associated with obesity (dyslipidemia, diabetes, high blood pressure) and breast tumor feature (size, multifocality, inflammatory breast cancer, lymph node invasion).

For analyses, patients were divided into age group intervals of 10 years or into four groups according to their BMI by using the World Health Organization classification of underweight (BMI < 18.5 kg/m^2^), normal weight (BMI 18.5–24.9 kg/m^2^), overweight (BMI 25–29.9 kg/m^2^), and obese (BMI ≥ 30 kg/m^2^).

### 2.2. Fatty Acid Profile of Adipose Tissues

As previously described [14], total lipids were extracted and triglycerides were isolated by preparative thin-layer chromatography (Millipore, Guyancourt, France). Fatty acids were methylated by using boron trifluoride (Sigma-Aldrich, Saint-Quentin Fallavier, France), and fatty acid methyl esters were analyzed by capillary gas chromatography (GC-2010 Plus chromatograph, Shimadzu, France) with a polar column (BPX70 column, cat#054623, SGE Analytical Science, Courtaboeuf, France). Fatty acid levels are expressed as percentage of the total integrated area. In this study, long-chain fatty acids between 20 and 24 carbons were included. Thus, long-chain saturated fatty acids (LC-SFAs) included 20:0, 21:0, 22:0, 23:0 and 24:0; long-chain monounsaturated fatty acids (LC-MUFAs) included 20:1n-9, 22:1n-9 and 24:1n-9; long-chain PUFA n-6 (LC-PUFA n-6) included 20:2n-6, 20:3n-6 (dihomo gamma lineloic acid (DGLA)), 20:4n-6 (arachidonic acid (AA)), 22:2n-6 and 22:4n-6, and long-chain PUFA n-3 (LC-PUFA n-3) 20:3n-3, 20:5n-3 (eicosapentaenoic acid (EPA)), 22:5n-3 (docosapentaenoic acid (DPA)) and 22:6n-3 (docosahexaenoic acid (DHA)).

### 2.3. Statistical Analysis

Statistical analyses were performed with Statview 4.57 (Abaccus Concepts, Berkeley CA, USA). For categorical variables (demographic variables, such as menopausal status, dyslipidaemia, diabetes and high blood pressure), patient number and percentage of the population were reported and the chi-square test was used. The continuous variables (age, BMI, fatty acid levels) were summarized with mean SD (in tables) or SEM (in Figures). To compare fatty acid level according to age or BMI categories, ANOVA followed by Fisher’s least significant difference test for multiple comparisons was used with 1 reference subgroup (i.e., 40–49 years age category or normal BMI category). To analyze the association between fatty acid content and age or BMI, we used Pearson’s correlation analysis and a correlogram designed using R 4.1.1 and RStudio 2021.09.0 available online (R Project, Free Software Foundation’s GNU project, Lucent Technologies, Murray Hill, NJ, USA, r-project.org, 25 August 2021). *p* ≤ 0.05 was considered statistically significant.

## 3. Results

This section is divided by subheadings. It should provide a concise and precise description of the experimental results, their interpretation, as well as the experimental conclusions that can be drawn.

### 3.1. Characteristics of the Population

The mean age of the population was 56.8 ± 13.8 years and mean BMI 25.4 ± 5.1. Among the 261 women, 12 (4.6%) were underweight, 123 (47.1%) were of normal weight, 76 (29.1%) were overweight and 50 (19.2%) were obese. 

The characteristics of the population, according to BMI categories, are shown in Table 1. Overweight and obese women were older (mean age about 59 years old, *p* = 0.01) than other women and, therefore, included a higher proportion of postmenopausal women (about 70%, *p* = 0.01). Pathologies associated with age and obesity were also more frequent, especially in obese women, with high blood pressure (48%, *p* = 0.01) and diabetes (18%, *p* = 0.0006).

### 3.2. Increase in LC-PUFA n-6 and LC-PUFA n-3 Content in Breast Adipose Tissue by Age in Normal-Weight Women

**Figure 1:**
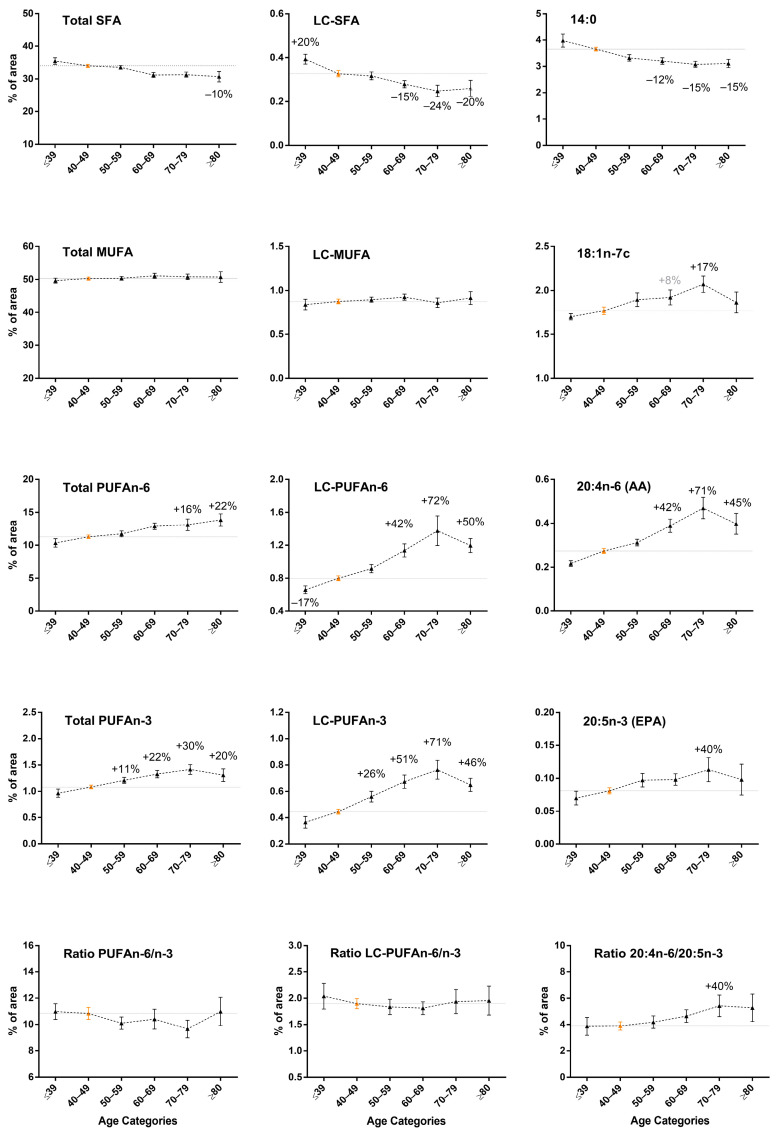
Changes in fatty acid content by age categories in normal-weight women. We divided 123 women with normal weight (body mass index (BMI) = 18.5–24.9 kg/m^2^) according to their age (≤39, 40–49, 50–59, 60–69, 70–79 and ≥80 years, with *n* = 10, *n* = 39, *n* = 28, *n* = 23, *n* = 16 and *n* = 7, respectively); fatty acid levels are expressed as mean ± SEM. Statistical analysis: ANOVA followed by Fisher’s LSD test for multiple comparisons using a reference subgroup (i.e., 40–49 years) for comparison with other age categories; significant variations >10% are specified in black above or below the curve (*p* ≤ 0.05). Variations close to significance (*p* ≤ 0.10) are in gray. SFA: saturated fatty acids, MUFA: monounsaturated fatty acids, PUFA: polyunsaturated fatty acids, LC: long chain fatty acids between 20 and 24 carbons, 14:0: myristic acid, 18:1n-7c: vaccenic acid, 20:4n-6: arachidonic acid [AA], 20:5n-3: eicosapentaenoic acid [EPA].

We first noted modifications in the fatty acid composition of breast adipose tissue according to age for women with normal BMI only (18.5–24.9 kg/m^2^) (Figure 1 and Table 1). With 40–49 years of age as a reference group for statistical analysis and considering only significant variations >10%, post-menopausal women showed major modifications in fatty acid composition for PUFAs, in particular, LC-PUFAs (from 20 to 24 carbons), which gradually increased with age. 

As compared with women aged 40–49 years, those aged 60–69 and 70–79 years showed the most increased 20:4n-6 (AA) proportion, from 42% and 71%, and similar modifications were observed for LC-PUFA n-6 (from 42% to 71% increase). Because 20:4n-6 (AA) and LC-PUFA n-6 represented <10% of PUFA n-6 content, the total PUFA n-6 content was slightly increased from +16% to +22% in older women. Similarly, LC-PUFA n-3 content showed a sharp and graduated increase, from 26% to 71%, according to age categories, especially 22:5n-3 (DPA, from +28% to 87% increase) and 22: 6n-3 (DHA, from 28% to 81% increase). Significant increases in LC-PUFA n-3 content were observed in women from 50 years of age. Of note, essential fatty acid levels (18:2n-6, linoleic acid and 18:3n-3, alpha linolenic acid that cannot be endogenously synthesized and directly comes from food) were only slightly modified with age, in particular, for 18:2n-6 (+12% and +20% in older women) (Table S1). Nevertheless, the PUFA n-6/n-3 and LC-PUFA n-6/n-3 ratios appeared stable across age categories (bottom of Figure 1), which indicates a common evolution. We noted a significant increase (+40%) in the 70–79 years-of-age category for the 20:4n-6/20:5n-3 ratio because 20:5n-3 rates did not change as sharply as 20:4n-6 rates across the ages.

All of these LC-PUFA gains were mainly at the expense of SFAs, 14:0, 18:0 and LC-SFAs, with significantly decreased contents from −10% to −24% in older women (from age 60, according to age category). Total SFAs were slightly but significantly affected as well, with a decrease of <10%. MUFA content was marginally altered by age, and only the level of 18:1n-7c was slightly increased, only in women from the 70–79 years group (+17%). 

In brief, in women with normal BMI, age led to fatty acid profile changes in breast adipose tissue, with a major increase in LC-PUFA content from both n-6 and n-3 series, at the expense of SFAs. Essential fatty acid levels remained relatively stable, which may suggest that age led to a regulation of fatty acid metabolism in favor of PUFA elongation, at the expense of SFA elongation.

### 3.3. Increase in LC-PUFA n-6 According to BMI but Not LC-PUFA n-3 Content in Breast Adipose Tissue

**Figure 2:**
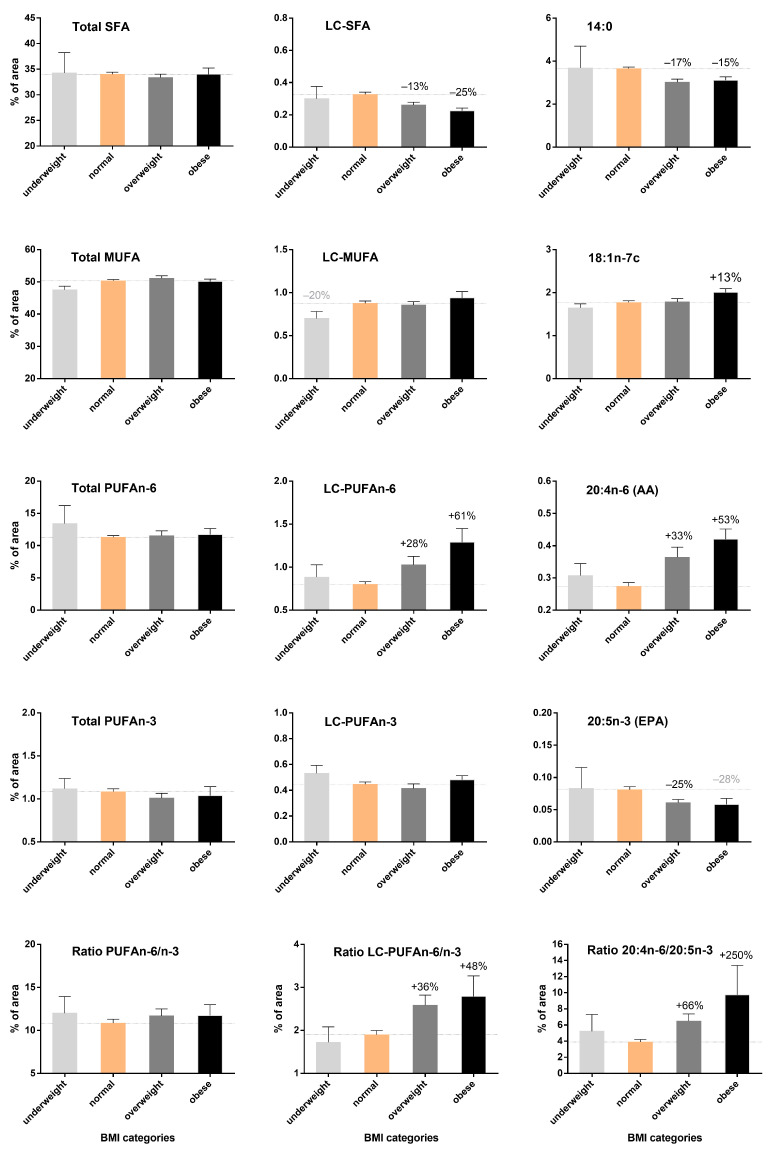
Changes in fatty acid content according to BMI categories in women aged 40–49 years. The 60 women were divided according to BMI categories (underweight: BMI < 18.5 kg/m^2^, normal: BMI 18.5–24.9 kg/m^2^, overweight: BMI ≥ 25 kg/m^2^, and obese: BMI ≥ 30 kg/m^2^, with *n* = 3, *n* = 39, *n* = 12, *n* = 6, respectively) and fatty acid levels expressed as mean ± SEM. Statistical analysis: ANOVA followed by Fisher’s LSD test for multiple comparisons using a reference subgroup (i.e., normal category) for comparison with other BMI categories; significant variations >10% are specified in black above the graph bar (*p* ≤ 0.05). Variations close to significance (*p* ≤ 0.10) are in gray.

To overcome the age effect, we evaluated the BMI impact on fatty acid profile for each age category. Fatty acid profiles in relation to BMI were fully presented only for women aged 40–49 years (Figure 2 and Table S2). 

For the 40–49 years population, considering only significant variations >10% and in comparison to normal-bodyweight women, we found essential fatty acid levels (18:2n-6 and 18:3n-3) not being significantly modified by BMI (Table S2). Nonetheless, the results showed a sharp increase in content of 20:4n-6 (AA) and LC-PUFA n-6, with +33% and +28% in the overweight group and + 53% and +61% in the obese group, respectively. Although the obese group showed an increase of +33% in 22:5n-3 (DPA) content and a not significant decrease (−28%) in 20:5n-3 (EPA) content, LC-PUFA n-3 content remained stable across BMI groups. These modifications were at the expense of some SFAs, such as 14:0 (−17% and −15%) and LC-SFAs (−13% and −25%) in overweight and obese groups, respectively. The obese group showed a slight increase (−13%) for 18:1n-7 in MUFA content.

Thus, overweight and obesity led to a gradual change in the fatty acid profile, with a sharp increase in LC-PUFA n-6 content, especially 20:3n-6, 20:4n-6 (AA) and 22:4n-6. Contrary to age, BMI was not associated with increased LC-PUFA n-3 content. This imbalance was not well accounted for by the standard PUFA n-6/n-3 ratio, which remained stable across BMI categories. Nevertheless, both LC-PUFA n-6/n-3 and 20:4n-6/20:5n-3 ratios (bottom of Figure 2) could account for this imbalance because they were largely and significantly increased in overweight women (+36% and +66%, respectively) and obese women (+48% and +250%), respectively.

### 3.4. Remodeling of the Fatty Acid Profile Induced by BMI Differs from Age-Induced Changes

**Figure 3:**
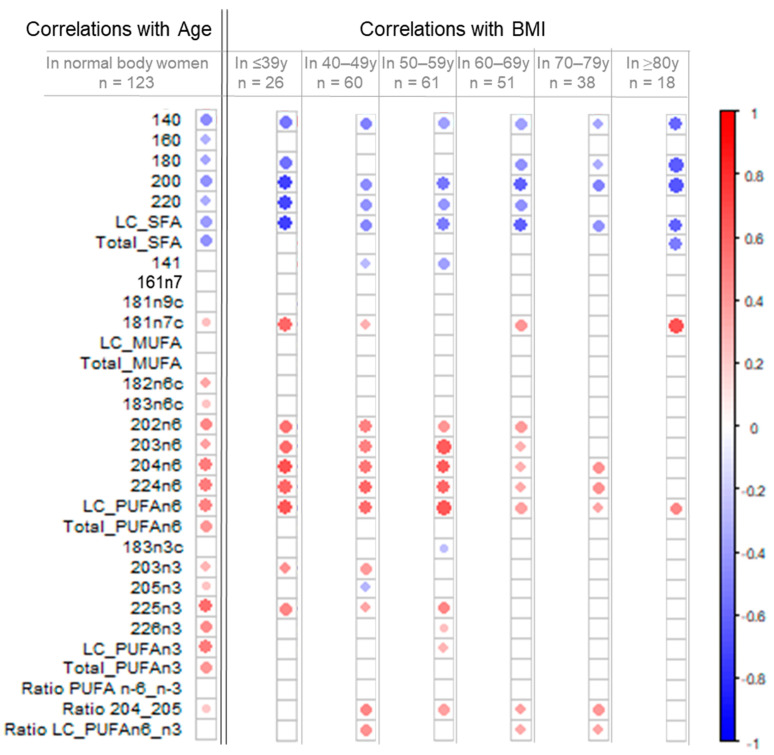
Correlations between fatty acid content and age or BMI. Left part of the figure: Correlogram between fatty acid content and age of 123 women with normal weight. Right part of the figure: Correlograms between fatty acid content and BMI with each age category (≤39 years: *n* = 26, 40–49 years: *n* = 60, 50–59 years: *n* = 68, 60–69 years: *n* = 51, 70–79 years: *n* = 38, ≥80 years: *n* = 18). Statistical analysis: correlation test (Pearson). The color intensity and size of the point is proportional to the absolute value of the correlation coefficient, so the stronger the correlation (i.e., the closer to −1 or 1), the darker and larger the point. The color legend shows a negative correlation (blue color) when the two variables varied in opposite directions, and a positive correlation (red color) when the two variables varied in the same direction. Only significant correlations (*p* < 0.05) are indicated by a point; otherwise, the box is empty. SFA: saturated fatty acids, MUFA: monounsaturated fatty acids, PUFA: polyunsaturated fatty acids, LC: long-chain fatty acids between 20 and 24 carbons.

To summarize and compare the variation in fatty acid content induced by age and BMI, we used a correlation analysis. To visualize these associations, we used the correlogram representation in Figure 3 (raw data, Table S3). The intensity of the color is proportional to the absolute value of the correlation coefficient, so the stronger the correlation (i.e., the closer to −1 or 1), the darker and larger the points. The color legend shows a negative correlation when the two variables varied in opposite directions (blue color) and a positive correlation when the two variables varied in the same direction (red color). Only significant correlations (*p* < 0.05) are shown by a point; otherwise, the box is empty.

To isolate the remodeling of the fatty acid profile induced by age, without BMI interference, correlation coefficients between fatty acid and age were calculated for the normal-bodyweight population (*n* = 123, left column, Figure 3 and associated table, Table S3). Thus, the content of both LC-PUFA n-6 and n-3 were positively correlated with age (increased with age); examples are 20:4n-6 (r = +0.50, *p* ≤ 0.0001), LC-PUFA n-6 (r = +0.48, *p* ≤ 0.0001), 22:6n-3 (r = +0.45, *p* ≤ 0.0001) and LC-PUFA n-3 (r = +0.50, *p* ≤ 0.0001), whereas several SFAs, mainly 14:0 (r = −0.42, *p* ≤ 0.0001), 20:0 (r = −0.41, *p* ≤ 0.0001) and LC-SFA (r = −0.35, *p* ≤ 0.0001), were negatively correlated with age (decreased with age). In MUFAs, only 18:1n-7c showed a moderate correlation with age (r = +0.25, *p* ≤ 0.01). 

To isolate the profile of fatty acid remodeling induced by BMI, without age interference, correlation coefficients between fatty acid content and BMI were calculated by using sub-populations from each age category (right part of Figure 3 and Table S3). Between ≤39y and 60–69y age categories, weight gain was correlated with a sharp increase in total LC-PUFA n-6 content, and mostly for 20:3n-6, 20:4n-6 and 22:4n-6, and to a decrease in the content of some SFAs, such as 14:0, 20:0 and LC-SFA. However, these correlations between BMI and the fatty acids constituting LC-PUFA n-6 series were less marked in older women because only LC-PUFA n-6 content remained significantly correlated with BMI in the ≥80 year category. Unlike LC-PUFA n-6, fatty acids of LC-PUFA n-3 series were correlated with BMI in only a few cases, in particular, for 22:5n-3 (DPA) in the first three age categories (i.e., ≤39, 40–49 and 50–59 years). 

Our results show that LC-PUFA n-6 and n-3 content together increased with age, particularly after menopause (from age 60 years). By contrast, weight gain led to an additional enrichment of LC-PUFA n-6 (20:2n-6, 20:3n-6, 20:4n-6 and 22:4n-6) in adipose tissue, with only a moderate addition of 22:5n-3 (DPA) in pre- and newly menopausal women (≤39, 40–49 and 50–59 years categories). These results suggest that an increase in BMI in pre- (≤39 years), peri- (40–49 and 50–59 years) and confirmed menopause (60–69 years) led to a prevalence of LC-PUFA n-6 content in adipose tissue, in addition to the changes already induced by age.

The imbalance in the n-6/n-3 ratio linked to the BMI increase was not reported by the standard PUFAs n-6/n-3 ratio (no correlation with BMI), mainly because LC-PUFAs n-6 represents only 8% to 10% of all PUFAs n-6, and LC-PUFA n-3, 40% to 45% of all PUFAs n-3. Two other ratios may be proposed to report this imbalance: 20:4n-6/20:5n-3 and LC-PUFA n-6/n-3. Although the 20:4n-6/20:5n-3 ratio was slightly increased with age, it seemed to capture the imbalance well and changed positively and significantly with BMI in four age categories (between 40–49 and 70–79 years), which included peri- and postmenopausal women. The LC-PUFA n-6/n-3 ratio could also have some relevance, but our results may show a lack in the age category 50–59 years (+0.21, *p* = 0.06).

### 3.5. Associations between Obesity and Indicators of Breast Tumor Aggressiveness

To explore the associations between indicators of tumor aggressiveness, fatty acid profile and weight gain, we first separated the population into the four BMI groups (Table S4). BMI increase appeared to be associated with tumor size (*p* = 0.16), lymph node involvement (*p* = 0.12), inflammatory breast cancer (*p* = 0.16) or tumor grade (*p* = 0.17), although the correlation did not achieve significant values. We then separated the population into groups of obese and non-obese women (Table 2).

As expected, our results show major regulations of long-chain fatty acids in obese versus non-obese women, with −44% of LC-SFAs (<0.0001), +38% of LC-PUFA n-6 (<0.0001), +35% of 20:4n-6 (AA), but a slight increase in LC-PUFA n-3 (+11%, *p* = 0.05) and no modification in LC-MUFA content. The standard PUFA n-6/n-3 ratio was non-discriminatory (*p* = 0.82), whereas the imbalance between LC-PUFA n-6 and n-3 in obese women was captured by both LC-PUFA n-6/n-3 (*p* = 0.002) and 20:4n-6/20:5n-3 ratios (*p* < 0.0001). 

Concerning indicators of tumor aggressiveness, tumor size (*p* = 0.23), the presence of tumor multifocality (*p* = 0.79), tumor grade (*p* = 0.54) and distribution of tumor phenotypes (*p* = 0.94), these were not associated with obesity. However, two worse prognostic indicators, axillary lymph node involvement (*p* = 0.02) and inflammatory breast cancer (*p* = 0.08), were more frequent in obese than non-obese women. Carcinoma in situ (a pre-invasive stage of breast cancer) associated with tumors was also less frequent in obese than non-obese women (*p* = 0.02). These results suggest that the very high BMI (state of obesity) could be a major factor favoring lymph node invasion and the appearance of the inflammatory breast cancer phenotype.

## 4. Discussion

Our results show that the age factor profoundly influenced the fatty acid profile in the adipose tissue of women. The changes mainly involved an increase in content of both LC-PUFA n-6 (including 20:3n-6 (DGLA), 20:4n-6 (AA), and 22:4n-6 (DTA)) and LC-PUFA n-3 (including 20:5n-3 (EPA), 22:5n-3 (DPA), and 22:6n-3 (DHA)) at the expense of some SFAs (mostly LC-SFAs). On analyzing the fatty acid profile in each age category, we found strong positive relations between BMI and content of LC-PUFA n-6 (including 20:3n-6 (DGLA), 20:4n-6 (AA), and 22:4n-6 (DTA)) but not LC-PUFA n-3. In contrast to age, the increase in BMI resulted in an imbalanced LC-PUFA n-6/n-3 ratio. Finally, axillary lymph node involvement and inflammatory breast cancer, both indicators of tumor aggressiveness, were more frequent in obese than non-obese women.

Most studies of fatty acid profiles in obese women have focused on circulating fatty acids or fatty acid profile in red blood cells. A meta-analysis showed significantly increased content of 20:3n-6 (DGLA) but not 20:4n-6 (AA) in the overweight/obese group, as compared with controls [24]. A few scattered data concerned adipose tissue and reported the obesity impact in non-cancerous patients. In a male/female adolescent population (*n* = 88), 20:4n-6 (AA) content was the best indicator explaining BMI variance in adolescents [25], and in male/female adults from Costa Rica, 20:4n-6 (AA) content was an independent marker of BMI and metabolic syndrome [26]. Our data for breast cancer patients broadly fit with these patterns that linked overweight/obesity and LC-PUFA n-6 and 20:4n-6 (AA) enrichment in breast adipose tissue.

Numerous nutritional intervention studies have shown that the fatty acid composition of adipose tissue is influenced by lipids provided in the diet [27], including in breast adipose tissue [28]. Adipose tissue could be proposed as a long-term biomarker of qualitative intake of dietary lipids [27]. Strong 20:4n-6 (AA) and LC-PUFA n-6 enrichment in adipose tissue of overweight/obese women may originate in food intake. Nevertheless, some studies did not find any significant association between 20:4n-6 (AA) intake (assessed by a food frequency questionnaire) and adipose 20:4n-6 (AA) content [26,29]. Some discrepancies exist, and the strongest correlations between nutritional lipids and adipose fatty acid content may be preferentially attributed to essential fatty acids (18:2n-6 and 18:3n-3) or best described for PUFA n-3 with a marine origin [27,30], whereas other fatty acids can also undergo endogenous metabolism. The potential remodeling in adipose tissue, induced by both age and weight gain, should be taken into account in women.

Our results suggest that the metabolism of fatty acids is in favor of PUFA elongation, at the expense of SFA elongation across age, namely after menopause. They are in agreement with those obtained by Bolton-Smith and co-workers [19]. An upregulation of some desaturases and elongases, especially those whose specificities were described for LC-PUFA synthesis (desaturases FADS1 and 2 and elongases ELOVL2 and 5), may explain fatty acid remodeling. However, studies focusing on the elongation capacities (especially conducted on LC-PUFAn-3) in humans showed i/ an extremely limited conversion of essential fatty acid to LC-PUFA in adults [31], ii/ the elongation/desaturation capacities seem to be estrogen dependent in women, as the limiting step of delta-6 desaturation (catalyzed by FADS2 enzyme) decreases after menopause [19] and capacities are restored by hormone replacement therapy [32]. Several other levels of regulation of desaturase have been identified and include food quality and composition [33] or genetic polymorphism of enzymes [34,35,36]. Altered circulating ELOVL2/5 elongase activity has been observed in patients with a metabolic syndrome related to overweight/obesity [37]. To date, the potential modification of elongase/desaturase activity linked to age or BMI needs further exploration in adipose tissue. To explain LC-PUFA accumulation in elderly women, alternative hypotheses may be an increase of LC-PUFA incorporation [38] and homeostasis changes through the related factor of lipid metabolism [39]. The process of fatty acid mobilization from or uptake by adipose tissue may be another reason for establishing specific accumulation of some fatty acids. In women, a metabolic shift in adipose tissue was especially observed with sex-hormonal disturbance during the menopausal transition, predisposing them to gain body fat [39,40,41,42]. In obese individuals, the storage function of adipose tissues seemed to be disturbed and depend on adipose anatomical location [43,44]. Moreover, some studies reported a certain selectivity of fatty acids when they enter or leave adipose tissue. MUFAs (namely 18:1n-9, oleic acid) and LC-PUFA n-6 are preferentially taken up by adipose tissue after a meal [45], but LC-PUFA n-3 (as 20:5n-3) and LC-PUFA n-6 (as 20:4n-6) are the first mobilized fatty acids during lipolysis [46]. With both weaker uptake and higher mobilization, adipose tissue may be easily depleted in PUFA n-3 content versus other fatty acids. These works need to be completed to identify the origins of the specific fatty acid composition linked to age or overweight/obesity in adipose tissue. 

**Figure 4:**
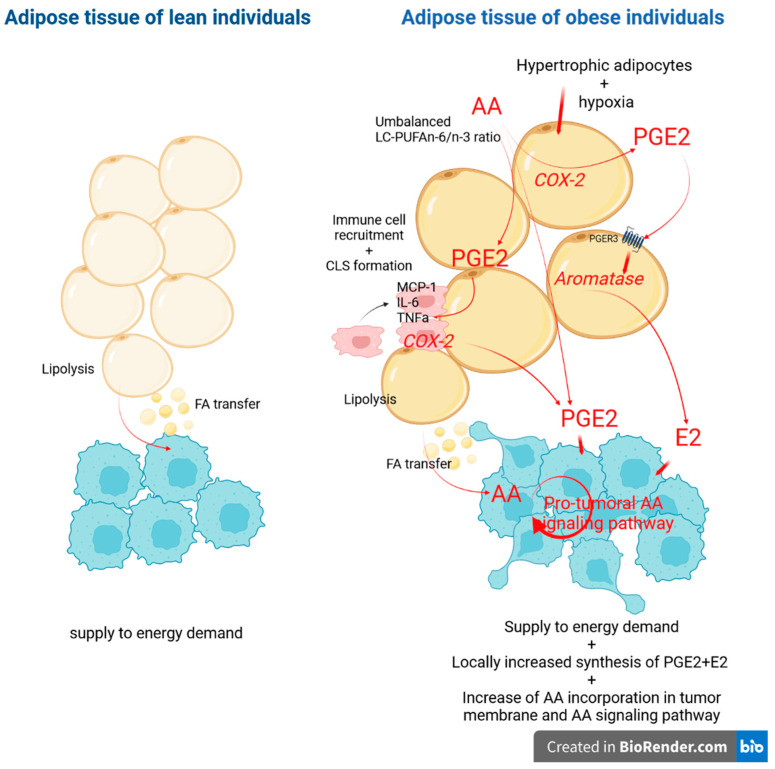
Hypothetical scheme: How can arachidonic acid (AA) accumulation in adipose tissue of obese individuals support tumor development and aggressiveness? In a lean individual, adipose tissue supplies to energy demand of tumor by adipocyte lipolysis and FA (Fatty Acid) transfer. In obesity context, the increased AA level of adipose tissue can potentiate the synthesis of PGE2 (by adipocytes and macrophages) and E2 (by adipocytes) providing molecules conducive to tumor proliferation and migration/invasion. Moreover, adipocyte lipolysis increases AA transfer to tumor cells that leads to an AA enrichment of tumor cells and their phospholipid membrane which feeds the pro-tumor AA signaling pathway. All words or arrows in red show an increase in molecules expression or activated/potentiated mechanism. AA: Arachidonic acid; COX-2: Cycloxygenase2; PGE2: Prostaglandine E2; E2: Estradiol; PGER3: Prostaglandine receptor 3; CLS: Crown-Like Structures, FA: Fatty acids. Based on knowledge provided by [50,51,52,53].

Some studies reported a relation between overweight/obesity and breast cancer recurrence or overall survival [2,3,4]. Our results in a small population show that obese patients more frequently had or tended to have two aggressiveness indicators: axillary lymph node involvement and inflammatory breast cancer. These results agreed with a previous retrospective study of 1599 breast cancer patients, conducted in our hospital [47]. In our study, adipose tissue showed increased LC-PUFA n-6 content, especially AA, without a counterbalance by PUFA n-3 content in obese women. As a substrate of cyclooxygenases, 5-lipoxygenase and P450 cytochrome enzymes, the 20:4n-6 [AA] is at the origin of eicosanoids synthesis, and the best availability of the substrate may lead to the preferential synthesis of pro-inflammatory eicosanoids, which could be a significant support for obesity-linked inflammation onset and tumor progression [48,49]. In Figure 4, we have included a hypothetical scheme to illustrate how AA accumulation in adipose tissue of obese individuals could affect tumor aggressiveness. In agreement with the involvement of this inflammatory process, non-steroidal anti-inflammatory drugs may reduce breast cancer recurrence in obese women by 50% [2].

Finally, obesity is generally associated with metabolic dysfunction and cardiometabolic disease that could originate in inflammation status of the adipose tissue and accumulation of visceral adipose tissue [54]. However, it is not uncommon to find a subset of individuals with metabolically healthy obesity (MHO), whose definition has not yet been fully standardized [55]. One might imagine that the greater the LC-PUFAn-6/n-3 imbalance, the greater the number of markers of metabolic disorder and adipose tissue inflammation. However, only further explorations could reveal relationships between BMI, fatty acid profile, metabolic disorder factors, and markers of adipose tissue inflammation. In addition, one may ask whether the prevalence of LC-PUFA n-6 induced by weight gain could be reversed by weight loss or whether LC-PUFA n-3 supplementation may reverse or mitigate LC-PUFA n-6-linked mechanisms. Weight loss could, at least in part, rebalance the fatty acid profile, because the mobilization of fatty acids does not depend on their proportion in adipose tissue, but rather on their structure (length and unsaturation number). PUFAs, such as 20:5n-3 and 20:4n-6, are more easily mobilized [56,57]. Moreover, several studies have described the benefit of PUFA n-3 in reducing inflammatory syndrome in the obesity context [58,59] and PUFA n-3 supplementation has been proposed in the prevention and treatment of breast cancer [11,60,61,62]. 

Limits of this study may be a lack of statistical power because of the number of individuals in certain sub-groups (youngest, oldest and thinnest women and inflammatory breast cancer case). The age pyramid of the population is adequate with the period of maximal incidence of breast cancer, but further exploration is needed on fatty acid profile and weight gain in the youngest women, namely premenopausal women. As a second limitation, only women were enrolled and only breast adipose tissue was explored. Hence, results cannot be generalized to men and to other adipose locations. Finally, specific ratios, such as LC-PUFA n-6/n-3 or 20:4n-6/20:5n-3, may be used to capture weight-gain-linked imbalance in adipose tissue. A lack of statistical significance was noticed in some subgroups (≤39, ≥80 years) but a PUFA n-3 diet supplementation that has not been brought to our attention could disturb our correlations between BMI and ratios. These ratios need to be validated in other populations.

## 5. Conclusions

Our results showed that weight gain in pre-, peri- and confirmed menopausal women led to an LC-PUFA n-6 and 20:4n-6 [AA] prevalence in adipose tissue. This accumulation could be of multifactorial origin, including an imbalanced diet, a modification of endogenous lipid metabolism (linked or not to genetics) and the delicate balance between the specific influx/efflux of some fatty acids in adipose tissue. Accumulation of the arachidonic acid in the adipose environment might explain how low-grade inflammation can set in in overweight/obese women, as described in epidemiological studies, and promote tumor emergence or progression. These findings could have public health implications, with regard to obesity and secondary/tertiary prevention of breast cancer via dietary and lifestyle interventions.

## Figures and Tables

**Table 1:** Characteristics of women with breast cancer by body mass index (BMI) category (*n* = 261).

BMI Category					
	Underweight BMI < 18.5 kg/m^2^ n = 12	Normal Weight BMI 18.5–24.9 kg/m^2^ n = 123	Overweight BMI 25–29.9 kg/m^2^ n = 76	Obese BMI ≥ 30 kg/m^2^ n = 50	*p* Value
Age (years), mean (SD)	47.7 (14.2)	55.7 (13.2)	58.6 (13.2)	59.4 (15.1)	0.01
BMI, mean (SD)	17.3 (1.5)	21.9 (1.6)	26.8 (1.4)	33.5 (3.4)	<0.0001
Menopause	5 (41.6%)	68 (55.2%)	53 (69.7%)	37 (74.0%)	0.02
High blood pressure	2 (16.7%)	32 (26.0%)	20 (26.0%)	24 (48.0%)	0.01
Diabetes	0 (0%)	2 (1.6%)	5 (6.6%)	9 (18.0%)	0.0006
Dyslipidemia	1 (8.3%)	30 (24.4%)	24 (31.6%)	19 (38.0%)	0.09

Data are *n* (%) unless indicated. HBP: high blood pressure; BMI: body mass index.

**Table 2:** Demographic characteristics, fatty acid content of adipose tissue and tumor aggressiveness indicators of women with breast cancer who were classified by their non-obese and obese status (*n* = 261).

	Non-Obese n = 211	Obese n = 50	*p* Values
Age (years)	56.3 (13.5)	59.4 (15.1)	0.15
BMI (kg/m²)	23.4 (3.2)	33.5 (3.4)	<0.0001
**Fatty acids content**			
LC-SFA	0.28 (0.09)	0.19 (0.06)	**<0.0001**
LC-MUFA	0.88 (0.17)	0.90 (0.17)	0.57
20:4n-6 (arachidonic acid)	0.37 (0.14)	0.50 (0.16)	**<0.0001**
LC-PUFA n-6	1.07 (0.42)	1.48 (0.41)	**<0.0001**
LC-PUFA n-3	0.59 (0.25)	0.66 (0.22)	**0.05**
**Ratios**			
PUFA n-6/n-3	10.75 (3.4)	10.87 (3.2)	0.82
20:4n-6/20:5n-3	5.02 (2.93)	7.30 (4.20)	**<0.0001**
LC-PUFA n-6/n-3	1.99 (0.76)	2.36 (0.73)	**0.002**
**Tumor phenotype**			0.94
Luminal A	72 (34.1%)	16 (32%)	
Luminal B	59 (45.5%)	16 (32%)	
HER2	28 (9.1%)	7 (14%)	
Triple negative	51 (9.1%)	11 (22%)	
UK	1 (0.5%)	-	
**Tumor grade**			0.54
1	17 (8.1%)	6 (12.0%)	
2	95 (45.0%)	23 (46.0%)	
3	96 (45.5%)	19 (38.0%)	
UK	3 (1.4%)	2 (4.0%)	
**Tumor size**	28.2 (18.7)	31.7 (19.3)	0.23
**Multifocal tumor**	50 (23.7%)	11 (22%)	0.79
**Axillary positive lymph node**	94 (44.5%)	31 (62.0%)	**0.02**
**Inflammatory breast cancer**	19 (9.0%)	9 (18.0%)	**0.08**
**Carcinoma in situ**	122 (57.8%)	20 (40%)	**0.02**

HBP: high blood pressure; BMI: Body mass index; LN: Lymph node; SFA: saturated fatty acids, MUFA: monounsaturated fatty acids, PUFA: polyunsaturated fatty acids, LC: long chain fatty acids between 20 and 24 carbons. UK: Unknown.

## Data Availability

Not applicable.

## Supplementary Materials

The following supporting information can be downloaded at: https://www.mdpi.com/article/10.3390/biomedicines10050995/s1, Table S1: Fatty acid composition of mammary adipose tissue in breast cancer patients with normal body mass index (18.5–24.9 kg/m2) according to age categories (*n* = 123); Table S2: Fatty acid composition of mammary adipose tissue in breast cancer patients according to body mass index categories in patients aged 40–49 years (*n* = 60); Table S3: Correlation coefficients between fatty acid contents and age or body mass index (BMI) in mammary adipose tissue of breast cancer patients. Table S4: Demographic and histological characteristics of breast cancer women (*n* = 261) according to BMI categories.
