# Supplementary material for: Accumulation of Arachidonic Acid, Precursor of Pro-Inflammatory Eicosanoids, in Adipose Tissue of Obese Women: Association with Breast Cancer Aggressiveness Indicators
Source: Biomedicines. 2022 Apr 26;10(5):995. doi: 10.3390/biomedicines10050995 (PMC9138452; doi:10.3390/biomedicines10050995)
Supplement: Supplementary file 1 [file biomedicines-10-00995-s001.zip › biomedicines-1668670-supplementary.pdf]

Table S1: Fatty acid composition of mammary adipose tissue in breast cancer patients with normal body mass index (18.5-24.9 kg/m^2) according to age categories (n = 123).

		Age categories																	
		≤39 years n = 10		40–49 years n = 39		50–59 years n = 28			60–69 years n = 23			70–79 years n = 16			≥80 years n = 7		
**Fatty acids** †		Mean	SD	Mean	SD	Mean	SD		Mean	SD		Mean	SD		Mean	SD	
**Saturated**																	
Myristic acid	14:0	3.98	0.78$^{\$}$	3.65	0.44	3.32	0.66*		3.20	0.59**	↘	3.08	0.45***	↘	3.11	0.41*	↘
Palmitic acid	16:0	23.74	2.18	23.35	1.80	23.28	1.97		21.72	2.35**		22.41	2.87		21.64	3.08$^{\$}$	
Stearic acid	18:0	6.63	1.07	6.00	1.23	5.99	1.19		5.36	1.19*	↘	4.98	0.87**	↘	5.03	1.41*	↘
LC-SFAs		0.39	0.07*	0.33	0.09	0.32	0.09		0.28	0.08*	↘	0.25	0.11**	↘	0.26	0.10$^{\$}$	↘
Total SFAs		35.48	3.19	34.00	2.73	33.53	2.96		31.22	3.68***		31.33	3.17**		30.70	4.23*	
**Mono-unsaturated**																	
Myristoleic acid	14:1	0.31	0.10	0.31	0.11	0.28	0.09		0.27	0.09		0.29	0.08		0.29	0.15	
Palmitoleic acid	16:1n-7c	3.34	0.86	3.66	1.50	3.81	1.24		3.71	1.19		4.33	1.20$^{\$}$		3.80	1.69	
Oleic acid	18:1n-9c	43.05	2.27	43.29	1.83	43.05	2.00		43.88	2.70		42.82	2.88		43.50	3.35	
Vaccenic acid	18:1n-7c	1.70	0.12	1.77	0.26	1.90	0.41		1.92	0.41$^{\$}$		2.07	0.37**	↗	1.86	0.32	
LC-MUFA		0.84	0.19	0.88	0.17	0.90	0.15		0.93	0.17		0.86	0.22		0.92	0.19	
Total MUFA		49.68	2.36	50.33	2.45	50.38	2.93		51.12	3.71		50.81	3.31		50.75	4.23	
**Polyunsaturated**																	
Linoleic acid	18:2n-6c	9.62	1.90	10.42	1.58	10.75	2.41		11.69	2.09*		11.67	3.42$^{\$}$	↗	12.57	2.55*	↗
γ Linolenic acid	18:3n-6	0.04	0.00	0.05	0.02	0.05	0.02		0.05	0.01		0.06	0.02*		0.05	0.01	
Eicosadienoic acid	20:2n-6	0.21	0.06$^{\$}$	0.24	0.05	0.26	0.05		0.28	0.05**		0.30	0.06***		0.29	0.03*	
Dihomo-γ-linoleic acid	20:3n-6	0.14	0.04	0.16	0.05	0.19	0.09$^{\$}$		0.25	0.13$^{\$}$		0.35	0.34*		0.27	0.08	
Arachidonic acid	20:4n-6	0.22	0.04	0.27	0.07	0.31	0.08		0.39	0.15***	↗	0.47	0.20****	↗	0.40	0.13**	↗
Docosatetraenoic acid)	22:4n-6	0.08	0.03	0.11	0.05	0.14	0.09		0.20	0.10***		0.25	0.16****		0.23	0.07**	
LC-PUFA n-6		0.66	0.15	0.80	0.18	0.92	0.27		1.13	0.38***	↗	1.38	0.72****	↗	1.20	0.22**	↗
**Total PUFA n-6**		10.35	2.00	11.30	1.30	11.74	2.39		12.90	2.27**	↗	13.13	3.46**	↗	13.85	2.49**	
Alpha linolenic acid	18:3n-3	0.60	0.15	0.64	0.16	0.65	0.21		0.64	0.22		0.65	0.18		0.66	0.27	
Eicosatrienoic acid	20:3n-3	0.03	0.01	0.03	0.01	0.04	0.01$^{\$}$		0.04	0.01*		0.04	0.01*		0.04	0.01	
Eicosapentaenoic acid	20:5n-3	0.07	0.03	0.08	0.03	0.10	0.05		0.10	0.04		0.11	0.07*	↗	0.10	0.06	
Docosapentaenoic acid	22:5n-3	0.14	0.05	0.18	0.06	0.23	0.09*	↗	0.29	0.11****	↗	0.33	0.12****	↗	0.28	0.07**	↗
Docosahexaenoic acid	22:6n-3	0.12	0.06	0.16	0.05	0.20	0.10*	↗	0.25	0.12***	↗	0.28	0.13****	↗	0.23	0.07*	↗
LC-PUFA n-3		0.37	0.14	0.45	0.12	0.56	0.22*	↗	0.67	0.24****	↗	0.76	0.29****	↗	0.65	0.13*	↗
**Total PUFAn-3**		0.97	0.24	1.10	0.21	1.21	0.29$^{\$}$	↗	1.33	0.34**	↗	1.42	0.37***	↗	1.31	0.32$^{\$}$	↗

**Ratio**												
PUFA n-6/n-3	10.99	1.90	10.85	2.86	10.10	2.48	10.42	3.64	9.66	2.68	10.99	2.86
LC-PUFA n-6/n-3	2.04	0.77	1.90	0.60	1.83	0.76	1.81	0.58	1.94	0.92	1.96	0.72
20:4n-6/20:5n-3	3.87	2.15	3.89	1.96	4.19	2.47	4.64	2.32	**5.43**	**3.26***	5.28	2.76

† expressed as % area, data are mean (SD: standard deviation), SFA: saturated fatty acids, MUFA: monounsaturated fatty acids, PUFA: polyunsaturated fatty acids, LC: long chain fatty acids between 20 and 24 carbons. Women included in this study were divided into age categories (every 10 years), and fatty acid content was expressed as mean with SD. Statistical analysis: ANOVA followed by Fisher's LSD test for multiple comparisons using a reference subgroup (i.e., 40-49 year category) for comparison with other age categories and variation > 10% was highlighted by bold numbers and an up or down arrow for positive or negative variation compared to the reference subgroup.

$ 0.05<p<0.1, *p≤0.05, **p<0.01, ***p<0.001, ****p<0.0001

Table S2: Fatty acid composition of mammary adipose tissue in breast cancer patients according to body mass index categories in patients aged 40–49 years (n = 60).

	BMI categories									
**Fatty acids** †	Underweight n = 3		Normal n = 40		Overweight n = 12			Obese n = 6		
	Mean	SD	Mean	SD	Mean	SD		Mean	SD	
**Saturated**										
14:0	3.68	1.76	3.65	0.44	**3.02**	**0.51****	↘	**3.11**	**0.40***	↘
16:0	23.49	3.08	23.35	1.80	23.62	1.24		24.60	2.15	
18:0	6.07	1.82	6.00	1.23	5.87	1.02		5.38	0.71	
LC SFA	0.30	0.13	0.33	0.09	**0.26**	**0.06***	↘	**0.23**	**0.04****	↘
Total SFA	34.27	6.91	34.00	2.73	33.38	2.23		33.99	3.04	
**Mono-unsaturated**										
14:1	0.26	0.22	0.31	0.11	0.24	0.07*		0.24	0.09	
16:1n-7c	3.26	1.53	3.66	1.50	3.24	1.20		3.36	0.77	
18:1n-9c	41.24	3.57 $	43.29	1.83	44.62	1.78*		42.97	1.72	
18:1n-7c	1.65	0.16 $	1.77	2.67	1.78	0.26		**2.00**	**0.23***	↗
LC-MUFA	0.70	0.14$	0.88	0.17	0.86	0.14		0.94	0.18	
**Total MUFA**	47.54	2.00 $	50.33	2.45	51.11	2.79		50.09	1.86	
**Polyunsaturated**										
18:2n-6c	12.47	4.58$	10.42	1.58	10.45	2.42		10.34	2.24	
18:3n-6	0.05	0.01	0.05	0.02	0.04	0.01		0.05	0.01	
20:2n-6	0.23	0.08	0.24	0.05	0.26	0.05		**0.32**	**0.08****	↗
20:3n-6	0.22	0.10	0.16	0.05	**0.24**	**0.13****	↗	**0.29**	**0.12*****	↗
20:4n-6	0.31	0.07	0.28	0.07	**0.36**	**0.11****	↗	**0.42**	**0.08******	↗
22:4n-6	0.12	0.05	0.11	0.05	**0.16**	**0.08***	↗	**0.25**	**0.14******	↗
LC-PUFA n-6	0.82	0.22	0.82	0.22	**1.03**	**0.34***	↗	**1.29**	**0.39*****	↗
**Total PUFA n-6**	13.42	4.83$	11.30	1.63	11.55	2.56		11.71	2.28	
18:3n-3	0.59	0.17	0.64	0.16	0.60	0.14		0.56	0.20	
20:3n-3	0.03	0.01	0.03	0.01	0.03	0.01		**0.04**	**0.01***	
20:5n-3	0.08	0.06	0.08	0.06	**0.06**	**0.02***	↘	**0.06**	**0.02$**	
22:5n-3	0.19	0.05	0.18	0.06	0.18	0.07		**0.23**	**0.04***	↗
22:6n-3	**0.23**	**0.02***	0.16	0.05	0.14	0.05		0.15	0.03	
LC-PUFA n-3	0.50	0.11	0.45	0.12	0.42	0.12		0.48	0.08	
**Total PUFA n-3**	1.12	0.20	1.08	0.21	1.01	0.19		1.04	0.26	
**Ratio**										
PUFA n-6/n-3	12.00	3.32	10.85	2.86	11.69	2.80		11.73	3.08	
LC-PUFA n-6/n-3	1.72	0.62	1.90	0.60	**2.59**	**0.82****		**1.17**	**0.48****	
20:4n-6/20:5n-3	5.24	3.58	3.89	1.96	**6.48**	**3.10***		**9.74**	**8.93*****	

† expressed as % area, data are mean (SD: standard deviation), SFA: saturated fatty acids, MUFA: monounsaturated fatty acids, PUFA: polyunsaturated fatty acids, LC: long chain fatty acids between 20 and 24 carbons.

Women included in this study were divided by age categories (every 10 years), and fatty acid levels expressed as mean with SD. Statistical analysis: ANOVA followed by Fisher's LSD test for multiple comparisons using a reference subgroup (i.e., normal BMI category) for comparison with other BMI categories and variation > 10% was highlighted by bold numbers and an up or down arrow for positive or negative variation (respectively) compared to the reference subgroup.

On the right, correlation coefficient between BMI and each fatty acid level in adipose tissue

$ 0.05<p<0.1, *p<0.05, **p<0.01, ***p<0.001, ****p<0.0001

Table S3: Correlation coefficients between fatty acid contents and age or body mass index (BMI) in mammary adipose tissue of breast cancer patients

	**Correlation with age**		**Correlation with BMI**													
	In normal body women n = 123		≤39 years n = 26		40–49 years n = 60		50–59 years n = 68		60–69 years n = 51		70–79 years n = 38		≥80 years n = 18	
**Fatty acids** †	r	*p*	r	*p*	r	*p*	r	*p*	r	*p*	r	*p*	r	*p*
**Saturated**														
14:0	-0.42	****	-0.53	**	-0.48	***	-0.37	**	-0.38	**	-0.34	*	-0.59	**
16:0	-0.31	***	+0.14		0.17		+0.18		+0.17		+0.03		-0.26	
18:0	-0.36	****	-0.56	**	-0.22	$	-0.22	$	-0.43	**	-0.32	*	-0.63	**
20:0	-0.41	****	-0.72	****	-0.45	***	-0.54	****	-0.61	****	-0.48	**	-0.65	**
22:0	-0.32	***	-0.71	****	-0.42	***	-0.42	***	-0.43	***	-0.12		-0.14	
LC-SFA	-0.35	****	-0.74	****	-0.45	***	-0.48	****	-0.58	****	-0.40	*	-0.60	**
Total SFA	-0.43	****	-0.34	$	-0.11		-0.06		-0.14		-0.14		-0.52	*
**Monounsaturated**														
14:1	-0.08		-0.33		-0.27	$	-0.38	**	+0.01		-0.15		+0.03	
16:1n-7c	+0.15		-0.09		-0.13		-0.16		+0.21		-0.07		+0.26	
18:1n-9c	+0.05		+0.27		+0.21		-0.10		+0.12		+0.30	$	+0.33	
18:1n-7c	+0.25	**	+0.59	**	+0.31	$	+0.19		+0.41	**	+0.19		+0.67	**
LC-MUFA	+0.10		+0.20		+0.21		+0.08		-0.08		+0.04		-0.19	
Total MUFA	+0.13		+0.25		+0.14		-0.12		+0.20		+0.24		+0.36	
**Polyunsaturated**														
18:2n-6c	+0.33	***	+0.10		-0.05		+0.04		-0.13		-0.12		+0.07	
18:3n-6	+0.23	**	-0.19		+0.01		+0.04		+0.04		+0.04		-0.04	
20:2n-6	+0.44	****	+0.55	**	+0.50	****	+0.42	***	+0.40	**	+0.28	$	+0.32	
20:3n-6	+0.35	****	+0.57	**	+0.49	***	+0.63	****	+0.30	*	+0.17		+0.47	$
20:4n-6	+0.50	****	+0.67	***	+0.52	****	+0.63	****	+0.31	*	+0.43	**	+0.31	
22:4n-6	+0.49	****	+0.59	**	+0.60	****	+0.59	****	+0.33	*	+0.42	**	+0.40	$
LC PUFA n-6	+0.48	****	+0.64	***	+0.57	****	+0.64	****	+0.37	*	+0.34	*	+0.47	*
**Total PUFAn-6**	+0.39	****	+0.20		+0.03		+0.13		-0.07		-0.06		+0.11	
18:3n-3	+0.05		-0.27		+0.08		-0.25	*	-0.23		-0.17		+0.20	
20:3n-3	+0.29	**	+0.43	*	+0.39	**	+0.18		+0.23	$	+0.17		+0.39	
20:5n-3	+0.23	$	+0.04		-0.29	*	-0.18		-0.17		-0.18		-0.10	
22:5n-3	+0.56	****	+0.47	*	+0.36	**	+0.47	****	+0.14		+0.13		+0.28	
22:6n-3	+0.45	****	+0.29		-0.18		+0.24	*	-0.04		-0.10		+0.13	
LC-PUFA n-3	+0.50	****	+0.36	$	+0.05		+0.29	*	-0.02		-0.03		+0.18	
**Total PUFA n-3**	+0.40	****	+0.07		-0.03		+0.03		-0.13		-0.09		+0.27	
**Ratio**														
PUFA n6/n3	-0.07		+0.112		+0.06		+0.06		+0.08		+0.04		-0.21	
LC-PUFA n6/n3	-0.03		+0.22		+0.43	**	+0.21	$	+0.33	*	+0.34	*	+0.11	
20:4n6/20:5n3	+0.21	*	+0.29		+0.46	****	+0.37	**	+0.36	**	+0.41	**	+0.17	

† expressed as % area, SD: standard deviation, SFA: saturated fatty acids, MUFA: monounsaturated fatty acids, PUFA: polyunsaturated fatty acids, LC: long chain fatty acids between 20 and 24 carbons. To establish correlations between fatty acid content and age, only women with normal BMI were included (left column of table). To establish correlations between fatty acid content and

BMI, women were divided by age category (right columns of table).Statistical analysis: Pearson's test (with correlation coefficient r and p $0.05<p\leq0.1$, *$p<0.05$, **$p<0.01$, ***$p<0.001$, ****$p<0.0001$

Table S4: Demographic and histological characteristics of breast cancer women (n = 261) according to BMI categories.

	BMI categories				
	Underweight<br>n = 12	Normal<br>n = 123	Overweight<br>n = 76	Obese<br>n = 50	P value
	Mean (SD) or number (%)	Mean (SD) or number (%)	Mean (SD) or number (%)	Mean (SD) or number (%)	
Age (years)	47.2 (14.2)	55.7 (13.2)	58.6 (13.2)	59.3 (15.1)	**0.02**
BMI (kg/m²)	17.3 (1.5)	21.9 (1.6)	26.8 (1.4)	33.5 (3.4)	**<0.0001**
**Fatty acids**					
LC-SFA	0.34 (0.10)	0.31 (0.10)	0.23 (0.06)	0.19 (0.06)	**<0.0001**
LC-MUFA	0.84 (0.20)	0.89 (0.17)	0.88 (0.16)	0.90 (0.17)	0.72
20:4n-6 (arachidonic acid)	0.29 (0.12)	0.33 (0.13)	0.43 (0.14)	0.50 (0.16)	**<0.0001**
LC-PUFA n-6	0.86 (0.34)	0.98 (0.41)	1.26 (0.37)	1.48 (0.41)	**<0.0001**
LC-PUFA n-3	0.55 (0.25)	0.56 (0.23)	0.64 (0.28)	0.66 (0.22)	**0.03**
**Ratios**					
PUFA n-6/n-3	11.11 (3.08)	10.46 (2.84)	11.17 (4.23)	10.87 (3.22)	0.51
20:4n-6/20:5n-3	4.38 (2.40)	4.38 (2.42)	6.15 (3.40)	7.30 (4.20)	**<0.0001**
LC-PUFAn-6/n-3	1.81 (0.80)	1.89 (0.69)	2.19 (0.82)	2.36 (0.73)	**0.0005**
**Tumor phenotype**					
Luminal A	4 (36.3%)	47 (38.2%)	21 (27.6%)	16 (32.0%)	0.69
Luminal B	5 (45.5%)	29 (23.6%)	25 (32.3%)	16 (32.0%)	
HER2	1 (9.1%)	15 (12.2%)	12 (15.8%)	7 (14.0%)	
Triple negative	1 (9.1%)	32 (26.0%)	18 (23.7%)	11 (22.0%)	
UK	1 (9.1%)	-	-	-	
**Tumor grade**					
1	2 (16.6%)	6 (4.9%)	9 (11.8%)	6 (12.0%)	0.17
2	2 (16.6%)	62 (50.4%)	31 (40.8%)	23 (46.0%)	
3	7 (58.3%)	53 (43.1%)	36 (47.4%)	19 (38.0%)	
UK	1 (8.3%)	2 (1.6%)	-	2 (4.0%)	
**Tumor size**	22.58 (11.13)	26.65 (15.70)	31.22 (23.48)	31.56 (19.48)	0.16
**Multifocal tumor**	2 (16.36%)	28 (22.7%)	20 (26.3%)	11 (22%)	0.82
**Axillary positive LN**	4 (33.3%)	57 (46.3%)	33 (43.4%)	31 (62.0%)	0.12
**Inflammatory Breast cancer**	0 (0%)	10 (8.1%)	9 (11.8%)	9 (18%)	0.15
**With ductal carcinoma in situ**	10 (83.3%)	71 (57.8%)	41 (53.9%)	20 (40%)	**0.03**

HBP: high blood pressure; BMI: Body mass index; LN: Lymph node; SFA: saturated fatty acids, MUFA: monounsaturated fatty acids, PUFA: polyunsaturated fatty acids, LC: long chain fatty acids between 20 and 24 carbons. UK: Unknown
